# Water Quality Assessment and Evaluation of Human Health Risk in Mutangwi River, Limpopo Province, South Africa

**DOI:** 10.3390/ijerph18136765

**Published:** 2021-06-24

**Authors:** Rofhiwa T. Madilonga, Joshua N. Edokpayi, Elijah T. Volenzo, Olatunde S. Durowoju, John O. Odiyo

**Affiliations:** 1 Department of Hydrology and Water Resources, University of Venda, Thohoyandou 0950, South Africa; madilongarofhiwateresa@gmail.com (R.T.M.); volenztom@gmail.com (E.T.V.); olatunde.durowoju@univen.ac.za (O.S.D.); johno@vut.ac.za (J.O.O.); 2 Office of the DVC Research Innovation Commercialization and Internationalization (RICI), Vaal University of Technology, Vanderbijlpark 1911, South Africa

**Keywords:** land use activities, Mutangwi River, water quality, *E*. *coli*, carcinogenic risk, water quality index

## Abstract

Freshwater supply is essential to life on Earth; however, land use activities such as mining and agriculture pose a significant danger to freshwater resources and the wellbeing of aquatic environments. This study temporarily assesses the water quality characteristics of Mutangwi River. Physicochemical parameters (pH, temperature, total dissolved solids (TDS), salinity, electrical conductivity (EC), and turbidity) were determined in situ using an Extech multimeter and turbidity meter. The concentration of the selected metals (Mg, Cr, Fe, Cd, Mn, Pb, Ca, and Na) were analysed using an Atomic Absorption Spectrophotometer. Membrane filtration method was used to analyse microbiological parameters (*Escherichia coli* and *Enterococci*). The physicochemical water quality parameters as well as basic anions (fluoride, phosphate, sulfate, nitrate, and chloride) determined complied with the regulatory guideline of the World Health Organization (WHO) and the South Africa National Standards (SANS). Some of the trace metals (Mn, Ca, Fe, and Mg) were found below the guideline values, while others (Pb and Cd) exceeded the threshold limit. The counts for *E*. *coli* (814.5–2169 cfu/100 mL) and *Enterococci* (333–9396 cfu/100 mL) in the study did not comply with the regulatory guidelines. The water quality status using the water quality index (WQI) indicated that on the average, the water quality from Mutangwi River is poor (WQI > 100). The hazard quotient through ingestion exposure did not exceed the threshold limit of 1, for adults and children. This implies that there is no potential non-carcinogenic health risk from trace elements via ingestion of drinking water for children and adults. However, cancer risk for adults and children was computed in relation to Cd and Pb levels and exceeded the threshold limit 10^−4^, indicating a possible carcinogenic risk. Water from the river should be adequately treated prior to domestic and agricultural use.

## 1. Introduction

Water quality often determines the fitness of water use for a variety of purposes. The assessment of water quality is thus important to evaluate the water use potential of any water resource [1]. The consumption of clean and safe water has been linked to increased health outcomes globally [2,3]. Despite the achievements that have been recorded with increased access to potable water, millions of people suffer various health-related preventable diseases due to the consumption of contaminated water [4].

In many cities and towns globally, sustainable access to clean and safe water has been reported; however, unfortunately, many people who live in peri-urban and rural regions of the world do not have continuous access to clean and safe water and they often resort to several alternative sources for their domestic water needs [5,6,7,8,9]. In most low-income countries, water is usually abstracted from rivers, lakes, and dams and used without any form of treatment [10,11]. The ease of access to surface water is one of the controlling factors contributing to its wide use, though it is often prone to higher chances of contamination and serves as one of the major sinks for environmental pollution [10,12]. Water quality is of a great concern globally because the decline in its quality due to contamination has great economic and public health burden [5,7,10].

In South Africa, surface waters such as rivers, lakes, ponds, and streams are key components for water supply due to the numerous dams that are fed from them. In the absence of sustainable access to potable water in rural areas, people are left to seek for alternative sources to meet their basic needs, and surface water is the first point of call as it is easy to access and use [2,3,11]. Consumption of untreated and inadequately treated water remains a major disease burden to public health and causes waterborne diseases such as cholera, typhoid, and dysentery [10,12]. Furthermore, high levels of trace metals and anions in drinking surface water have been reported to cause various health complications, including gastric cancer, baby blue syndrome, altered reproduction potential, and mouth ulceration [13,14].

Anthropogenic and natural factors can cause an increase in the level of contamination of freshwater sources [8,15]. Activities such as human settlements, industrialization, and agriculture (crop and livestock farming) have adversely affected the quality of most rivers, streams, and dams. Although there are legislations to protect surface water bodies from pollution, such as the pollutant pay principle, they are hardly enforced [5]. This has consequently led to increased contamination of surface water bodies, which are often a source of domestic water, agricultural water, occupation, and recreation to many who lives along their course [9].

Mutangwi River is widely used for domestic, recreational, and agricultural purposes (irrigation and animal watering). Moreover, small-scale businesses such as car washes abstract water from the river. Small-scale fishing also occurs within the river course. Edokpayi et al. [12] reported the discharge of inadequately treated hospital wastewater effluent into the river before it joins the Nzhelele River, which feeds the Nzhelele dam for water supply. Other potential sources of pollution to the river system include open dumping of solid wastes, open grazing of free-ranging animals, and surface runoff from various farmlands within its course. Hence, it is of outmost importance to report on the water quality status of Mutangwi River as, to the best of our knowledge, there are no published data on this important water resource in a semi-arid region of South Africa.

We, therefore, report our findings on the physicochemical and microbiological characteristics of Mutangwi River. In addition, we present the overall status of water in the river using the Water Quality Index (WQI), which is a versatile tool for summarizing the water quality status of a river system. Furthermore, since the water is used for several purposes, we computed the potential carcinogenic and noncarcinogenic health risk associated with its consumption based on the trace metals levels recorded.

## 2. Materials and Methods

### 2.1. Study Area

**Figure 1:**
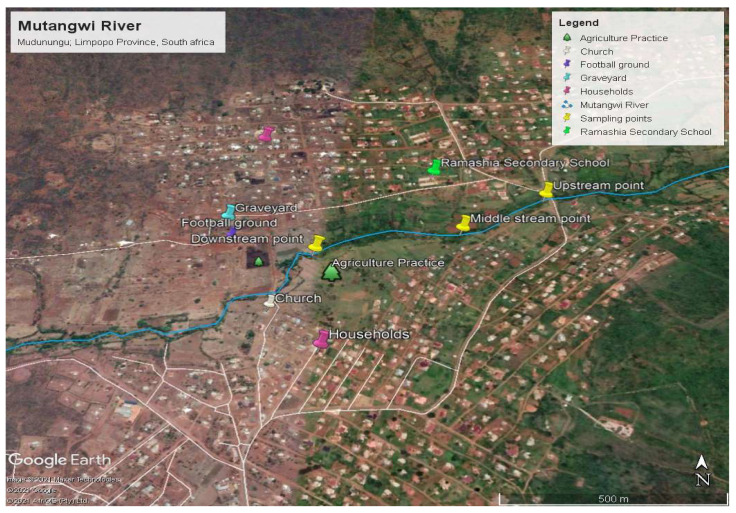
Map of the study area.

The study area is situated at Mudunungu Village, Thulamela Municipality. It is located at a height of 1318 m above the sea level. Its geographical location is 22°52′60′′ S and 30°13′0′′ E. Mutangwi River (Figure 1) is generally used for various activities but mainly for domestic and agriculture purposes (Thulamela Municipality IDP Review, 2013–2014). It is estimated that about 80% of rainfall is received in summer (September–March) compared to the winter period (April–August). Daily temperature in the catchment ranges between 12 and 22 °C in the dry season and between 20 and 41 °C during the wet season [16].

The mean yearly precipitation ranges from 480 to 560 mm, with a mean annual runoff of about 50 million cubic meters [16]. 

The potential pollution sources include ablution facilities, sanitation, car wash, laundry, and dumping of refuse on open spaces and on the riverbank. Land use activities vary in the upper, middle and lower parts of Mutangwi River. The river is covered by large-scale agricultural activities, there is dominant forest in the upper stream and the lower part has cropping plantation. 

### 2.2. Sampling

Mutangwi River was divided into three sections, which are the upstream, midstream, and downstream, for samples collection. Twenty-seven water samples were collected in triplicate using sterilized plastics bottles on a monthly basis between June and August 2018. Samples were collected for physicochemical, microbiological, and trace metals analyses. Water samples for metals were preserved with concentrated nitric acid. Collected samples were kept in an ice chest and transported to the Hydrology Laboratory of the University of Venda for further examination. Microbial analysis was performed within 6 h of sample collection.

### 2.3. Samples Analysis

#### 2.3.1. Physiochemical Parameters

Temperature, pH, electrical conductivity (EC), salinity, and total dissolved solids (TDS) were measured in the field using an Extech multimeter (EC 400, Extech Instruments, Nashua, NH, USA). Turbidimeter was used to measure the turbidity (TB 400, Extech Instruments, Nashua, NH, USA) of the water samples.

#### 2.3.2. Trace Metals

Trace metals were analysed using an atomic absorption spectrophotometer (900H, Perkin Elmer, Akron, OH, USA). Calibration standards were prepared from 100 mg/L stock solution of the test metals.

#### 2.3.3. Anions

Anion (fluoride, phosphate, sulphate, nitrate, and nitrite) concentrations were determined using Ion Chromatography (IC). The water samples were filtered with syringe filters (0.45 μm) and placed in a standard vial in an automated autosampler connected to the IC (850 professional IC, Metrohm AG, Herisau, Switzerland). 

#### 2.3.4. Microbiological Analysis

*Escherichiacoli* and *Enterococcus* levels in the samples were evaluated using Membrane Filtration (MF) technique. M-Tec Chromo select Agar (Sigma Aldrich, Johannesburg, South Africa) was prepared for *E*. *coli* enumeration while m-Enterococcus Agar (Sigma Aldrich, Johannesburg, South Africa) was used for the determination of *enterococci* levels. The agars were prepared according to the manufacturer’s guidelines. The samples (100 mL) were filtered through 47 mm sterile membrane filters using a vacuum pump and a manifold by adhering to the protocols of the American Public Health Association [17]. The filter paper containing the test organisms was incubated for 24 and 48 h at 37 °C and 45 °C for *E*. *coli* and *enterococci*, respectively. The results were reported as colony-forming units per 100 mL of sample.

### 2.4. Water Quality Index (WQI)

WQI is an index that reflects the composite impact of various water quality parameters for easy interpretation and use by water administrators. The WQI was computed through three steps. First, each of the 15 parameters was assigned a weight (*w_i_*) according to their relative importance in the overall quality of water for drinking purposes (Table 1). The maximum weight of 5 was assigned to a parameter because of its major importance in water quality assessment, minimum weight of 1 was assigned to those parameters deemed insignificant to the overall water quality. Other parameters were assigned weights between 1 and 5 based on their relative significance in the water quality evaluation. The mathematical formula used for the WQI computation is presented in the equations below [18,19]:

(1) $$Wi=\frac{wi}{\sum_{i=1}^{n}wi}$$

Wi is the unit weight of pollutant variable; n is the total number of pollutant variables; wi is the weight of each parameter.

(2) Quality rating scale (qi)=(CiSi)×100

qi is the quality rating, Ci is the concentration of each chemical parameter in each water sample in mg/L, and Si is the drinking water standard for each chemical parameter in mg/L.

For computing WQI, qi and Wi were used as shown in Equation (3) below [18,19].

(3) $$\mathrm{WQI}=\overset{n}{\underset{i=1}{\sum }}Wi\times qi$$

WQI ranks water quality in the range of excellent to unsuitable for drinking with numerical values computed using Equation (3) (Table 1).

### 2.5. Quantitative Health Risk Assessment

Human exposure risk pathways of an individual to trace metals contamination could be through three main pathways including inhalation via nose and mouth, direct ingestion, and dermal absorption through skin exposure. Common exposure pathways to water are dermal absorption and ingestion routes. Exposure dose for determining human health risk through these two pathways has been described in the literature [21] and can be calculated using the equations below:

(4) $$Exp_{ingestion}=\frac{C_{water}\times IR\times EF\times ED}{BW\times AT}$$

(5) $$Exp_{dermal}=\frac{C_{water}\times SA\times Kp\times ET\times IR\times EF\times ED\times CF}{BW\times AT}$$

where $Exp_{ingestion}$ is the exposure dose through ingestion of water (mg/kg/day); $Exp_{dermal}$ is the exposure dose through dermal absorption (mg/kg/day); $C_{water}$ is the average concentration of the estimated metals in water (µg/L), and Kp is the dermal permeability coefficient in water (cm/h): 0.001 for Cu, Mn, Fe, and Cd, while 0.0006 for Zn, 0.002 for Cr, and 0.004 for Pb. The other constants in those equations are shown in Table 2.

Potential noncarcinogenic risks due to exposure of trace metals were determined by comparing the calculated contaminant exposures from each exposure route (ingestion and dermal) with the reference dose (*RfD*). The constant of $RfD_{ingestion}$ of Cd, Pb, Mn, and Fe is 0.5, 1.4, 24, and 700, respectively, while $RfD_{dermal}$ values of analysed trace elements Cd, Pb, Mn, and Fe are 0.025, 0.42, 0.96, and 140 [21]. The Hazard Quotient (HQ) toxicity potential of an average daily intake to reference dose for an individual via the two pathways can be determined using Equation (6) [22].

(6) $$HQ_{ingestion/dermal}=\frac{Exp_{ingestion/dermal}}{RfD_{ingestion/dermal}}$$

where$\;RfD_{ingestion/dermal}$ is ingestion/dermal toxicity reference dose (mg/kg/day). The $RfD_{ingestion/dermal}\;$values were obtained from the literature [18,21,23]. An HQ < 1 is assumed to be safe and taken as significant noncarcinogenic [24], but HQ > 1 indicates a potential health risk to those exposed to the levels of the contaminant.

To assess the overall potential noncarcinogenic effects posed by more than one metal and pathway, the sum of the computed HQs across metals was expressed as hazard index (HI) [21]. HI > 1 showed that exposure could have a potential adverse effect on human health [18].

Carcinogenic risk (CR) through ingestion pathway was estimated using Equation (7):

(7) $$CR_{ingestion}=Exp_{ingestion}\times CSF$$

where $CR_{ingestion}$ is cancer risk through ingestion of trace metals-contaminated water, $Exp_{ingestion}$ is average daily dose (mg/kg/day) of heavy metals, and CSF is cancer slope factor (mg/kg/day). The slope factor for Pb and Cd is 0.009 and 6.1, respectively.

## 3. Results and Discussion

### 3.1. Physical Parameters

The EC values ranged from 182.25 to 233.17 µS/cm (Table 3). The values recorded complied with the standard guideline of <1700 mS/m [25] and 600 mS/m [26], respectively. The average values of TDS ranged from 126.37 to 167.5 mg/L, and were numerically higher in downstream sites (Table 3) and complied with regulatory standards.

Temperature plays a crucial role in water bodies with regards to chemical responses and the metabolic rates of life forms and it is therefore a controlling factor of aquatic species distribution [27,28]. Mean temperatures recorded in the sampling sites were within the recommended guideline set by SANS and WHO (25 °C) [25,26]. The temperature is suitable for use of irrigation (6.5–8.4) and aquaculture (6.5–9), respectively. The pH of the samples was in the range of 7.03–7.15 (Table 3) and complied with the recommended guidelines for human consumption [25,26]. Sudden changes in pH can have an adverse impact on aquatic biota.

Salinity level varied between 83.89 and 133.52 mg/L (Table 3). Excessive salinity may cause eye irritation in human and chlorosis in plants [29]. Mean values of salinity recorded complied with the standard guidelines [25,26]. Turbidity levels recorded were above the permissible limit of 1 and <5 NTU prescribed by SANS and WHO, respectively, for domestic water use in all sampling points. Average turbidity level of the study ranged from 3.87 to 5.54 NTU (Table 3). However, it was within the permissible limit of aquaculture water use (25 NTU) [28]. The level of turbidity was quite low compared to others reported in the region [30]. Comparable levels have been reported by Ejoh et al. [31] at Ubongo and Egini Rivers, Udu LGA, Delta State Nigeria (2.3–5.8 NTU).

### 3.2. Chemical Parameters

#### Major Cations and Anions

**Figure 2:**
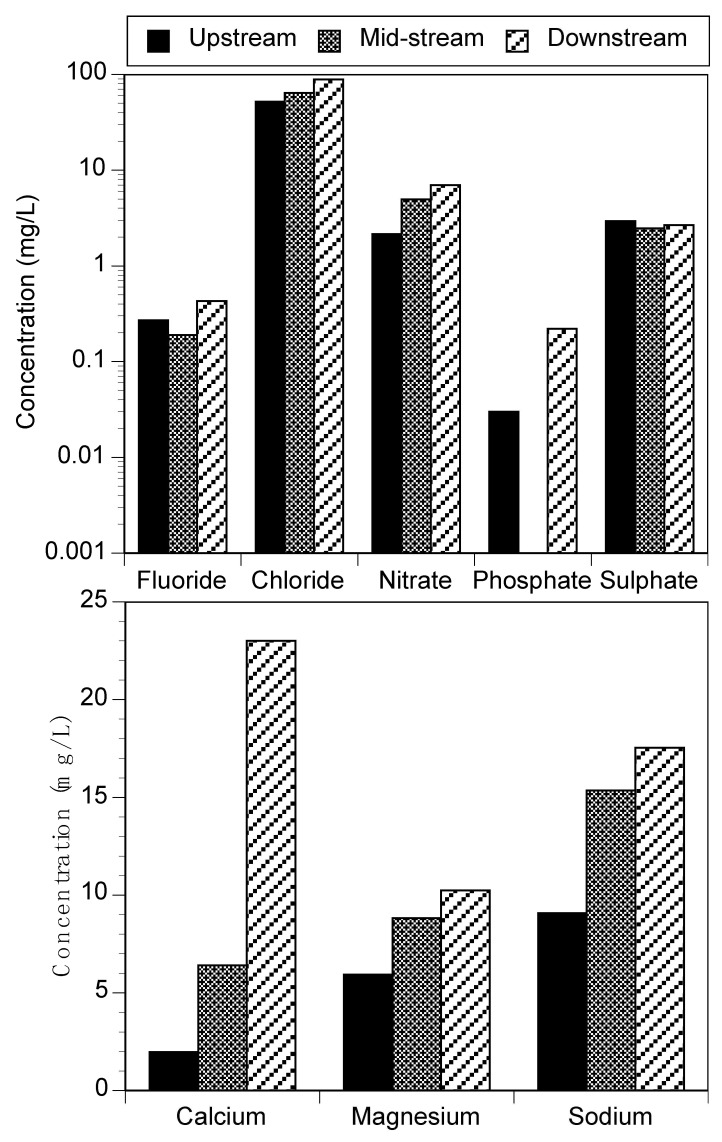
Major anions (**top**) and cations (**bottom**) in Mutangwi River.

Fluoride ($F^{-})$ is an essential anion in drinking water. Its occurrence in levels <0.5 mg/L has been linked with dental caries in children, while higher levels exceeding 1.5 mg/L can cause dental and skeletal fluorosis as well as non-fluorosis diseases [32,33]. Low levels of fluoride below 0.5 mg/L were determined in this study (Figure 2). The other anions (Cl^−^, NO_3_^−^, PO_4_^3−^, and SO_4_^2−^) were within the permissible limit for domestic and agricultural water use. The levels of nitrates and phosphates determined in this study can be linked with the practice of agriculture around the river course. Figure 2 shows that higher levels of the various anions were recorded at the downstream sampling sites than upstream, which could be due to increase in anthropogenic activities along the river course.

Concentration of phosphate recorded in all sampling points varied between 0.03 and 0.22 mg/L (Figure 2) and complied with the regulatory guidelines for domestic purposes, irrigation, and livestock water use. A study by Wei et al. [34]. observed that phosphate in water is not viewed as toxic directly to animals and people. However, its presence in high levels can initiate poisonous algal blooms and hypoxic waters with reduced biotic diversity. A study by Awomeso et al. [35] showed that the concentration of phosphates in Nairobi River ranged from 2.0 to 3.34 mg/L.

Sulphate (${\mathrm{SO}}_{4}^{2-})\;$is a crucial and essential nutrient for tissue development in plants and animals. Sulphate levels recorded ranged from 2.46 to 2.94 mg/L (Figure 2). Concentrations in all sampling points were within the limit of DWAF and WHO for residential water utilization of 200 and 250 mg/L, respectively [25,28]. Nitrogen is important to human health, but a high level in other food products and drinking water could lead to major and serious health problems. The concentration of nitrate varied between 2.15 and 6.98 mg/L (Figure 2) and complied with the WHO (50 mg/L) threshold limit for domestic water use [25]. High nitrate (${\mathrm{NO}}_{3}^{-}$) levels in drinking water can cause methemoglobinemia in infants [36,37].

The concentration of chloride ranged from 52.08 to 88.59 mg/L (Figure 2). Chloride (${\mathrm{Cl}}^{-})$ levels were higher compared with other anions examined in the study but complied with the guideline value for drinking water and agricultural purposes.

Calcium (Ca) is vital major cation for biochemical interactions in living organisms. The recorded levels varied between 1.96 and 23 mg/L (Figure 2) and complied with regulatory standards for domestic and agricultural water use [28]. Low levels of Sodium (Na) in the range of 9.08–17.55 mg/L (Figure 2) were determined in the study area and complied with several regulatory standards for domestic and agricultural water use. Similarly, low levels of magnesium were also determined in this study. Like the anions, higher levels were found at the downstream sites.

### 3.3. Trace Metals Concentration

The concentration of lead (Pb) ranged from 0.05 to 0.07 mg/L and exceeded the threshold limit of 0.01 mg/L for drinking water. The levels found are of health risk to humans and aquatic organisms. High levels of Pb above 0.01 mg/L have been linked to anaemia, memory loss, anorexia, brain damage, and death. Furthermore, this finding can be compared to studies reported by Ayandiran et al. [38] in Rupsha and Oluwa Rivers in South West Nigeria.

**Figure 3:**
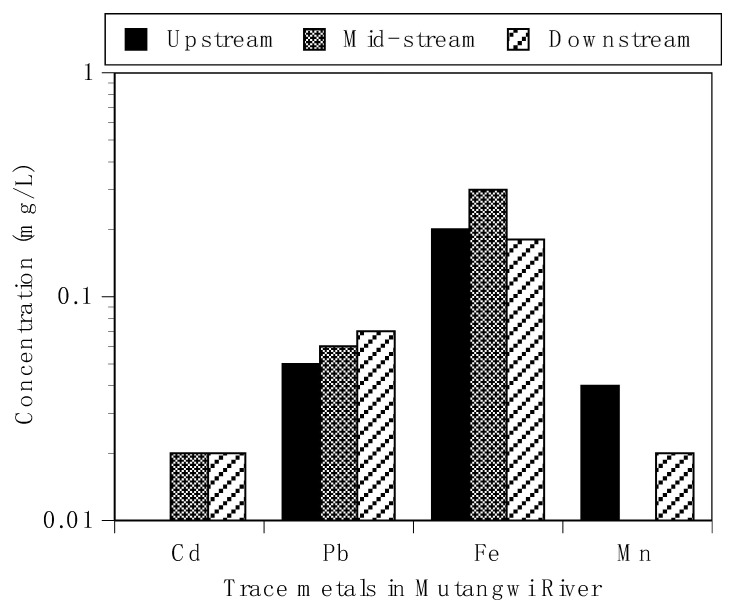
Trace metal concentration in Mutangwi River.

The levels of cadmium (Cd) determined in this study ranged between 0.01 and 0.02 mg/L and did not comply with safe levels as stipulated by WHO [25] and SANS [26] (Figure 3). The levels found could cause potential ecological risk of metals to aquatic organisms. Moreover, with respect to Cd, the water is not fit for aquaculture and irrigation of fresh vegetables. The presence of cadmium is a major concern since it can cause potential health risk to humans and aquatic organisms. The levels of cadmium recorded could be from emission through air and water from hazardous waste sites and factories. High levels of Cd have been linked to several diseases in man and aquatic organisms, including memory loss, reproductive defect, and cancer, as well as damage to the lungs, kidney, and immune system, which could eventually lead to death [39].

Iron is a metal at the dynamic site of numerous significant redox proteins in plants and animals [40]. The concentration of iron (Fe) ranged between 0.18 and 0.3 mg/L (Figure 3). The SANS guideline of 2 mg/L associated with chronic effect associated with Fe consumption via water was not exceeded in any of the sampling months. In addition, the aesthetic guideline value of 0.3 mg/L was not exceeded [25]. High levels of Fe have been associated with several anthropogenic activities such as washing of clothes and cars in rivers. Fe is known to affect the aesthetic property of water, interfering with the taste and appearance of water. The consumption of Fe-rich water has been implicated with negative effects on human health, such as hypertension, congestion of blood vessels, and increased respiration rate [41]. The levels of Mn ranged between 0.01 and 0.05 mg/L and complied with regulatory standards for drinking and irrigation of fresh vegetables. Both Fe and Mn can adversely affect the taste of water and influence the water aesthetic properties if their level exceeds the permissible limit [26].

### 3.4. Microbiological Parameters

**Figure 4:**
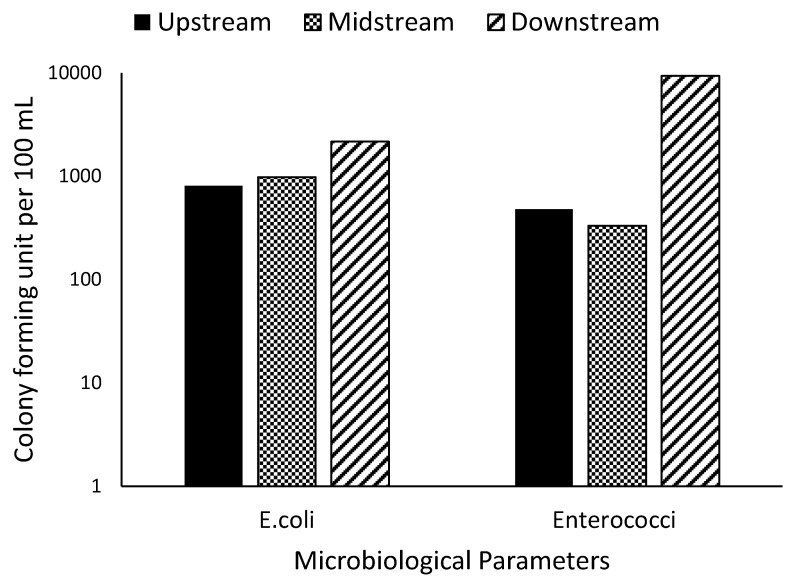
*E*. *coli* and *Enterococci* levels in the sampling sites.

The values of *E*. *coli* count were in a range of 814.5–2169 cfu/100 mL, and higher values were recorded at the downstream sampling sites of the river (Figure 4). This level exceeded the regulatory standards for drinking water (0 cfu/100 mL) [25,26] and agriculture use (irrigation) (1 cfu/100 mL) [28]. Mutangwi River is contaminated with *E*. *coli* and therefore is not suitable for irrigation, recreation, and domestic purposes without proper treatment. *Enterococci* levels differed distinctively in each of the sampling points. *Enterococci* is an opportunist pathogen whose occurrence in freshwater systems has been strongly linked to sewage discharge, and they usually show resistance to antibiotics [42]. They are often considered as a good indicator for the assessment of microbiological risks to humans and aquatic life [43]. *Enterococci* levels recorded in the study ranged from 333 to 9396 cfu/100 mL and did not comply with regulatory standards (Figure 4).

In various river systems in South Africa, different levels of microbial contamination have been recorded, which often necessitate the need for disinfection before use. Table 4 shows the levels of *E*. *coli* and *Enterococci* reported in some rivers in South Africa. The potential sources of pollution have been linked to surface runoffs, discharge of sewage water, open defecation by free-ranging animals, dumping of diapers by the river bank, etc. [4,44]. The consumption of faecal-contaminated water has been implicated in various disease outbreaks, such as diarrhoea and cholera. The consumption of raw vegetables irrigated with faecal-contaminated water in local areas has led to stomach cramps, vomiting, and diarrhoea [45].

### 3.5. Water Quality Index

Water Quality Index (WQI) is significant rating that determines the general overall water quality status in a singular term that is useful for the determination of suitable treatment and use [8,21,57]. One of the limitations of WQI is that it does not account for microbial water quality parameters. 

The WHO guideline for drinking water quality was used for the calculation of WQI. The rating of the river water was computed using the physicochemical parameters obtained. Table 5 shows how the WQI was determined.

Results from Table 5 show that the upstream of Mutangwi River has water of good quality that can be used for various purposes. However, the water quality of the midstream and downstream of the river is poor, and the overall rating of the water quality of the river is poor. Hence, the river water quality is poor both microbiologically and physicochemically and should not be used without appropriate treatment.

There was a strong correlation between nitrate and phosphate with a value of *r* = 0.74 (Table 6), inferring that they could be from similar sources such as sewage effluent, drainage from farmland, and fertilizers. A strong correlation exists between TDS and EC (*r* = 0.99), as expected, as they are both directly proportional to each other. The more solids that are dissolved in the water, the higher is the value of the electric conductivity. The sources of ions could be natural, i.e., geological condition, and from human activities such as domestic and industrial waste and also from agricultural activities. *E*. *coli* had a strong correlation with *Enterococci* with (*r* = 0.99) (Table 6). Hence, the presence of *E*. *coli* can be used to infer the presence of *Enterococci*. Pb showed a strong correlation to Cd (*r* = 0.86). Other correlation results can be seen in Table 6.

### 3.6. Human Health Risk Assessment

#### 3.6.1. Noncarcinogenic risk

Summary of Hazard Quotient (HQ) values for some trace elements (Pb, Cd, Mn, and Fe) in drinking water through ingestion and dermal routes were computed for adults and children (Table 7 and Table 8). The trace metals can pose potential adverse health effects when the HQ value of a metal is higher than 1 [58]. The HQ through ingestion and dermal exposure for both children and adult groups did not exceed 1 in all sampling points as well as the hazard index (HI). Hence, we did not find evidence for noncarcinogenic risk related to trace elements (Pb, Cd, Mn, and Fe) in Mutangwi River. The occurrence of acute illness, however, is expected due to the levels of *E*. *coli* and *enterococci* recorded. Similar findings have been reported in previous studies [59,60,61,62].

#### 3.6.2. Carcinogenic Risk (CR)

The cancer risk was computed based on the intake level of inorganic Pb and Cd, which may increase carcinogenic effects depending on the exposure dose and duration of exposure. Only metals that are carcinogenic in nature were used in this computation. Considering ingestion exposure pathways, estimated *CR_ingestion_* values for adults were in the range of (5.15 ×$\;10^{-5}\;$to 1.75 × $10^{-1}$) and were (1.31 × $10^{-1}\;$to 9.33 × $10^{-1}$) for children. The average values of $CR_{ing}$ for adults and children were 1.80 × $10^{-1}\;$and 9.89 × $10^{-1}$, respectively. Pb had the highest average contribution of CR compared to Cd. $CR_{ing}$ levels of Pb exceeded the threshold for both children and adults for all selected sampling points (Table 7 and Table 8). This result clearly shows that children are more vulnerable to health risks associated with drinking water than adults. Similar studies showing the vulnerability of children to chemical contaminants in food and water have been reported [63,64]. These findings imply that the water is of a poor quality and should be treated prior to domestic water use. The use of water with Pb and Cd levels higher than the permissible limit can also be of health risk to the fishes that live in the water.

## 4. Conclusions

The results obtained in this study have presented baseline data on the water quality of Mutangwi River. All the physicochemical parameters, except for turbidity, complied with regulatory standards, as did the levels of the anions. All the major cations also fell within the standard limit. Pb and Cd were present in elevated levels above the threshold limit of SANS and WHO. Similarly, the levels of faecal coliform bacteria recorded did not comply with regulatory standards. The downstream of the river was more contaminated compared to the upstream and midstream. The WQI showed that the quality of the upstream of the river can be regarded as good while the mid and downstream are poor and need treatment before use. Due to the levels of microbes in the water, the water quality of Mutangwi River can be regarded as poor, although it can be used for irrigation of tree crops. There was no potential noncarcinogenic risk (HQ < 1) associated with the consumption of the river water. However, carcinogenic risk was computed for both children and adults. Therefore, adequate treatment of water from this river is highly recommended.

## Figures and Tables

**Table 1:** Water quality rating based on WQI.

WQI	Quality Status	Possible Use	Grading
50	Excellent water quality	Domestic purposes	A
50–100	Good water quality	Any purpose without treatment	B
100–200	Poor water quality	Irrigation and partial body contact	C
200–300	Very poor water quality	Irrigation and industrial, domestic	D
>300	Unsuitable for drinking purposes	Proper treatment required before use	E

Adapted and revised from [20].

**Table 2:** Health risk assessment of different exposure through parameter.

Parameter	Unit	Child	Adult
Exposure Frequency (EF)	Day/year	365	365
Body Weight (BW)	kg	15	70
Ingestion Rate (IR) or Daily intake (DI)	L/day	1.8	2.2
Exposure Duration (ED)	Years	6	70
Skin surface Area (SA)	${\mathrm{cm}}^{3}$	6600	18,000
Exposure Time (ET)	Hours/day	1	0.58
Conversion Factor (CF)	$L/{\mathrm{cm}}^{3}$	0.001	0.001
Averaging Time (AT)	Days	365 × 6	365 × 70
Particular Emission Factor (PEM)	$m^{3}/\mathrm{kg}$	1.3 × 10^9^	1.3 × 10^3^

Adapted from [21].

**Table 3:** Mean concentrations and standard deviation of the physiochemical water quality parameters.

Parameters	Upstream Average and SD	Midstream Average and SD	Downstream Average and SD	WHO [25]	SANS [26]	Limit of Agriculture Water Use [28]		
						Livestock	Irrigation	Aquaculture
Temperature (°C)	18.18 ± 2.43	18.69 ± 1.44	18.08 ± 2.29	<25	<25			
pH	7.03 ± 0.13	7.15 ± 0.4	7.08 ± 0.19	6.5–8.5	6.5–9.5	n/a	6.5–8.4	6.5–9.0
Salinity (mg/L)	83.89 ± 25.15	110.67 ± 6.69	133.52 ± 45.04	600–900	<1500	n/a	n/a	n/a
TDS (mg/L)	126.37 ± 38.61	166.01 ± 7.07	167.5 ± 19.56	0–400	≤1200	0–3000	n/a	n/a
EC (µs/cm)	182.25 ± 51.65	235.83 ± 12.97	233.17 ± 2.51	600.01	≤1700	n/a	0–3000	n/a
Turbidity (NTU)	5.54 ± 0.50	3.87 ± 2.54	3.965 ± 1.73	<1	<1	n/a	n/a	25

TDS, total dissolved solids; EC, electrical conductivity; SD, standard deviation; n/a, guideline value not available.

**Table 4:** Comparison levels of *E*. *coli* and *Enterococci* in Mutangwi River and other rivers in South Africa.

Rivers	*E*. *coli* (cfu/100 mL)	*Enterococci* (cfu/100 mL)	Provinces	Year	References
Olifants River	34–1599	1620–2760	Mpumalanga	2012	[46]
Tyume River	100–16,000	33–5100	Eastern Cape	2013	[47]
Msunduzi River	1–39	1–79	Limpopo	2013	[48]
Buffalo River	0–190	0–5,300,000	KwaZulu Natal	2013	[49]
Klip and Vaal Rivers	>5	>40	Gauteng	2014	[50]
Eerste Rivers	5–100	17–510	Western Cape	2015	[51]
Mooi River	61–548	74–870	North West	2016	[52]
Mvudi River	1650–4767	950–11,533	Limpopo	2016	[40]
Apies River	3.9	3.97	Gauteng	2017	[53]
Luvuvhu River	>300–>400	19,100–25,000	Limpopo	2017	[45]
Mooder River	01–62	12–104	Free state	2018	[54]
Nzhelele River	100–57,000	100–80,000	Limpopo	2018	[6]
Mutoti River	29.2–57.1	20–2180	Limpopo	2020	[55]
Umhlangane River	246	377	KwaZulu Natal	2021	[56]
Mutangwi River	814.5–2169	333–9396	Limpopo	2021	Current study

**Table 5:** Physio-chemical parameters used for WQI determination.

Parameters	Desirable Limit (Si)	Weight of Each Parameter (wi)	Relative Weight (Wi)	qi upstream	qi Midstream	qi Downstream	WQI Upstream	WQI Midstream	WQI Downstream	WQI (Mean)
pH	6.5–8.5	4	0.0714	93.73	95.33	94.40	6.64	6.68	6.74	6.69
TDS (mg/L)	500	4	0.0714	25.27	33.20	33.5	1.83	2.37	2.34	2.18
EC (µs/cm)	600.01	4	0.0714	30.37	39.30	38.86	2.81	3.8	2.77	3.12
Salinity	600	3	0.0535	13.98	18.4	22.2	0.075	0.984	1.19	0.74
Temperature	25	2	0.0357	72.72	74.76	72.32	2.59	2.66	2.5	2.58
Fluoride (mg/L)	1.0	4	0.0714	27.0	19.0	43.0	1.92	1.35	3.0	2.09
Chloride (mg/L)	250	3	0.0535	20.83	25.72	35.44	1.11	1.38	21.89	8.13
Nitrate (mg/L)	45	5	0.0892	4.77	10.97	15.51	0.42	0.97	1.38	0.92
Sulphate (mg/L)	200	3	0.0535	1.47	1.23	1.34	0.15	0.126	0.14	0.14
Fe (mg/L)	0.3	3	0.0535	66.66	99.9	60	3.56	5.3	3.21	4.02
Ca (mg/L)	75	2	0.0357	2.68	8.5	10.27	0.09	0.30	0.36	0.25
Mg (mg/L)	30	2	0.0357	19.77	29.4	34.13	0.71	1.04	1.21	0.99
Na (mg/L)	100	3	0.0535	9.08	15.32	17.55	0.48	0.81	0.93	0.74
Pb (mg/L)	0.01	5	0.0892	500	600	700	44.6	53.52	62.44	53.52
Cd (mg/L)	0.005	5	0.0892	200	400	400	17.84	35.68	35.68	29.73
		∑wi=56	∑wi=1.00				∑WQI=84.82	∑WQI=116.97	∑WQI=145.78	115.86

**Table 6:** Statistical analysis of correlation result.

Parameters	Temp. (°C)	pH	Salinity	TDS	EC	Turb.	F^−^	Cl^−^	NO_3_^−^	PO_4_^3−^	SO_4_^2−^	*E*. *coli*	Enterococci	Fe	Mn	Pb	Ca	Mg	Na	Cd
Temp. (°C)	1																			
pH	0.83	1																		
Salinity (mg/L)	−0.11	0.45	1																	
TDS (mg/L)	0.33	0.79	0.9	1																
EC (µs/cm)	0.4	0.83	0.86	0.99	1															
Turb. (NTU)	−0.41	−0.84	−0.86	−0.99	−0.99	1														
F^−^ (mg/L)	−0.85	−0.41	0.61	0.22	0.14	−0.139	1													
Cl^−^ (mg/L)	−0.36	0.23	0.97	0.77	0.72	−0.72	0.78	1												
NO_3_^−^ (mg/L)	−0.06	0.49	0.99	0.92	0.88	−0.88	0.58	0.96	1											
PO_4_^3−^ (mg/L)	−0.72	−0.21	0.77	0.41	0.34	−0.34	0.97	0.89	0.74	1										
SO_4_^2−^ (mg/L)	−0.73	−0.98	−0.59	−0.88	−0.91	0.92	0.25	−0.39	−0.63	0.05	1									
*E*. *coli* (cfu/100mL)	−0.53	0.01	0.89	0.62	0.55	−0.55	0.9	0.97	0.87	0.97	−0.18	1								
*Enterococci* (cfu/100mL)	−0.64	−0.1	0.83	0.51	0.44	−0.44	0.94	0.93	0.8	0.99	−0.05	0.99	1							
Fe (mg/L)	0.99	0.83	−0.11	0.3	0.39	−0.4	−0.84	−0.33	−0.06	−0.71	−0.73	−0.53	−0.63	1						
Mn (mg/L)	−0.64	−0.95	−0.68	−0.93	−0.95	0.96	0.14	−0.5	−0.71	−0.06	0.99	−0.29	−0.17	−0.6	1					
Pb (mg/L)	−0.15	0.41	0.99	0.88	0.84	−0.83	0.65	0.98	0.99	0.79	−0.56	0.91	0.85	−0.2	−0.7	1				
Ca (mg/L)	0.15	0.66	0.96	0.98	0.96	−0.96	0.39	0.88	0.97	0.58	−0.78	0.75	0.66	0.14	−0.9	0.95	1			
Mg (mg/L)	0.04	0.58	0.98	0.95	0.93	−0.92	0.49	0.92	0.99	0.66	−0.71	0.82	0.74	0.03	−0.8	0.98	0.99	1		
Na (mg/L)	0.12	0.64	0.97	0.97	0.95	−0.95	0.42	0.89	0.98	0.6	−0.76	0.77	0.68	0.12	−0.8	0.96	0.99	0.99	1	
Cd (mg/L)	0.36	0.81	0.88	0.99	0.99	−0.99	0.18	0.75	0.9	0.39	−0.89	0.59	0.48	0.35	−0.9	0.86	0.97	0.94	0.96	1

Temp., temperature; Turb., turbidity; F^−^, fluoride; Cl^−^, chloride; NO_3_^−^, nitrate; PO_4_^3−^, phosphate; SO_4_^2−^, sulphate.

**Table 7:** Human health risk assessment indices for cancer risks from ingestion and absorption of studied metals for the adults.

Parameters	Sampling Points	$EXP_{ing}$	$EXP_{der}$	$HQ_{ing}$	$CR_{ing}$
	Upstream	$3.14\;\times \;10^{-4}$	$1.49\;\times \;10^{-6}$	$6.29\;\times \;10^{-4}$	5.15$\;\times \;10^{-5}$
Cd	Midstream	$6.29\;\times \;10^{-4}$	$2.98\;\times \;10^{-6}$	$1.26\;\times \;10^{-3}$	1.03$\;\times \;10^{-4}$
	Downstream	$6.29\;\times \;10^{-4}$	$2.98\;\times \;10^{-6}$	$1.26\;\times \;10^{-3}$	1.03$\;\times \;10^{-4}$
	Upstream	$1.57\;\times \;10^{-4}$	$2.98\;\times \;10^{-5}$	$1.12\;\times \;10^{-3}$	$1.75\;\times \;10^{-1}$
Pb	Midstream	$1.89\;\times \;10^{-4}$	$3.58\;\times \;10^{-5}$	$1.35\;\times \;10^{-3}$	$2.10\;\times \;10^{-1}$
	Downstream	$2.20\;\times \;10^{-4}$	$4.18\;\times \;10^{-5}$	$1.57\;\times \;10^{-3}$	$2.44\;\times \;10^{-1}$
	Upstream	$1.26\;\times \;10^{-3}$	$5.97\;\times \;10^{-6}$	$5.24\;\times \;10^{-5}$	-
Mn	Midstream	$3.14\;\times \;10^{-4}$	$1.49\;\times \;10^{-6}$	$1.31\;\times \;10^{-5}$	-
	Downstream	$6.29\;\times \;10^{-4}$	$2.98\;\times \;10^{-6}$	$2.62\;\times \;10^{-5}$	-
	Upstream	$6.29\;\times \;10^{-3}$	$2.98\;\times \;10^{-5}$	$8.98\;\times \;10^{-6}$	-
Fe	Midstream	$9.43\;\times \;10^{-4}$	$4.47\;\times \;10^{-5}$	$1.35\;\times \;10^{-5}$	-
	Downstream	$5.66\;\times \;10^{-3}$	$2.68\;\times \;10^{-5}$	$8.08\;\times \;10^{-6}$	-
	Upstream	-	-	$1.81\;\times \;10^{-3}$	-
HI	Midstream	-	-	$2.63\;\times \;10^{-3}$	-
	Downstream	-	-	$2.86\;\times \;10^{-3}$	-

HI, hazard index; -, not applicable.

**Table 8:** Human health risk assessment indices for cancer risks from ingestion and absorption of studied metals for the children.

Parameters	Sampling Points	$EXP_{ing}$	$EXP_{der}$	$HQ_{ing}$	$CR_{ing}$
	Upstream	$6.92\;\times \;10^{-3}$	$6.92\;\times \;10^{-3}$	$6.92\;\times \;10^{-3}$	$6.67\;\times \;10^{-1}$
Cd	Midstream	$1.00\;\times \;10^{-2}$	$1.00\;\times \;10^{-2}$	$1.00\;\times \;10^{-2}$	$1.31\;\times \;10^{-1}$
	Downstream	$1.09\;\times \;10^{-2}$	$1.09\;\times \;10^{-2}$	$1.09\;\times \;10^{-2}$	$9.33\;\times \;10^{-1}$
	Upstream	$6.92\;\times \;10^{-3}$	$6.92\;\times \;10^{-3}$	$6.92\;\times \;10^{-3}$	6.67 × 10^−1^
Pb	Midstream	$1.00\;\times \;10^{-2}$	$1.00\;\times \;10^{-2}$	$1.00\;\times \;10^{-2}$	$1.31\;\times \;10^{-1}$
	Downstream	$1.09\;\times \;10^{-2}$	1.09 × 10^−2^	$1.09\;\times \;10^{-2}$	$9.33\;\times \;10^{-1}$
	Upstream	$6.92\;\times \;10^{-3}$	6.92 × 10^−3^	$6.92\;\times \;10^{-3}$	-
Mn	Midstream	$1.00\;\times \;10^{-2}$	$1.00\;\times \;10^{-2}$	$1.00\;\times \;10^{-2}$	-
	Downstream	$1.09\;\times \;10^{-2}$	$1.09\;\times \;10^{-2}$	$1.09\;\times \;10^{-2}$	-
	Upstream	$6.92\;\times \;10^{-3}$	$6.92\;\times \;10^{-3}$	$6.92\;\times \;10^{-3}$	-
Fe	Midstream	$1.00\;\times \;10^{-2}$	$1.00\;\times \;10^{-2}$	$1.00\;\times \;10^{-2}$	-
	Downstream	$1.09\;\times \;10^{-2}$	$1.09\;\times \;10^{-2}$	$1.09\;\times \;10^{-2}$	-
	Upstream	-	-	$6.92\;\times \;10^{-3}$	-
HI	Midstream	-	-	1.00 × 10^−2^	-
	Downstream	-	-	1.09 × 10^−2^	-

HI, hazard index; -, not applicable.

## Data Availability

The data that support the findings of this study are available from the corresponding author, upon reasonable request.
